# Assessment of parasitological findings in heartworm-infected beagles treated with Advantage Multi® for dogs (10% imidacloprid + 2.5% moxidectin) and doxycycline

**DOI:** 10.1186/s13071-017-2190-9

**Published:** 2017-05-19

**Authors:** Molly D. Savadelis, Cameon M. Ohmes, Joe A. Hostetler, Terry L. Settje, Robert Zolynas, Michael T. Dzimianski, Andrew R. Moorhead

**Affiliations:** 1 University of Georgia, College of Veterinary Medicine, Athens, GA USA; 2 Bayer Animal Health, Shawnee, KS USA

**Keywords:** Canine heartworm disease, *Dirofilaria immitis*, Heartworm treatment, Macrocyclic lactone treatment, Moxidectin, Doxycycline, Advantage Multi®, Advocate®

## Abstract

**Background:**

Anecdotal reports support the position that the adulticidal heartworm treatment utilizing doxycycline and Advantage Multi®/Advocate® for Dogs (10% imidacloprid + 2.5% moxidectin) has successfully converted antigen-positive dogs to antigen-negative. To date, no controlled experimental studies have demonstrated the adulticidal efficacy of this treatment regimen. The aim of this study was to evaluate the parasitological and clinical efficacy of Advantage Multi® for Dogs (IMD + MOX) and doxycycline in heartworm-infected beagles.

**Methods:**

This study utilized 16 dogs, 8 dogs in each of non-treated control and treated groups. A total of 16 adult *Dirofilaria immitis* (Missouri strain) were surgically transplanted into the jugular vein of each study dog. The treatment regimen of monthly IMD + MOX topically (per labeled dosage and administration) for 10 months and 10 mg/kg doxycycline BID orally for 30 days was initiated 30 days post-surgical transplant. Echocardiograms, radiographs, complete blood counts, clinical chemistry profiles, heartworm antigenemia and microfilaremia were evaluated every 4 weeks. Serum samples were assayed for heartworm antigen using the DiroCHEK® heartworm antigen test. The DiroCHEK® was performed according to the manufacturer’s recommendations and read using a spectrophotometer at 490 nm.

**Results:**

All dogs tested positive for the presence of heartworm antigen post-surgical transplant and prior to treatment. Heartworm antigen levels began declining in treated dogs 3 months post-treatment. Non-treated control dogs remained antigen-positive. No microfilariae were detected in treated dogs after 21 days post-treatment. At necropsy, adult heartworms were recovered from all non-treated control dogs with a range of 10–12 adult worms/dog for an average recovery of 10.6 adult heartworms/dog. In the IMD + MOX- and doxycycline-treated dogs, the range of adult heartworms recovered was 0–2 adult worms/dog, with five dogs having no adult heartworms present. The average adult heartworm recovery was 0.6/dog in the treated group. This treatment regimen demonstrated a 95.9% efficacy in eliminating adult heartworms (*P* < 0.0001).

**Conclusions:**

This study demonstrated that this treatment regimen successfully eliminated *D. immitis* microfilariae by 21 days post-treatment, reduced heartworm antigen concentration over time, and had a 95.9% efficacy in the elimination of mature adult heartworms. Based on this study, we conclude that this treatment regimen is a relatively quick, reliable and safe option to treat canine heartworm infection as compared to other treatment regimens involving macrocyclic lactones, when the approved drug melarsomine dihydrochloride is unavailable, contraindicated or declined by an owner unable to afford the more costly treatment or concerned about the potential side effects.

## Background

Anecdotal reports from veterinarians describe the effectiveness of slow kill protocols in converting heartworm antigen positive canines to antigen negative. While slow kill protocols vary widely, the protocol can be described as the use of macrocyclic lactones at prophylactic doses with or without doxycycline to kill adult heartworms over an extended period of time [1, 2]. With the increasing popularity among veterinarians in utilizing slow kill adulticidal treatment for canine heartworm disease, experimental efficacy studies are necessary to determine if these protocols are effective.

Doxycycline administered at 20 mg/kg SID was shown to reduce the concentration of *Wolbachia* present in *D. immitis* in naturally infected dogs leading scientists to investigate the use of doxycycline during heartworm treatment [3]. Bazzocchi et al. [4] evaluated the use of weekly ivermectin (6 μg/kg) alone, doxycycline (10 mg/kg SID) alone, and a combination of ivermectin (6 μg/kg) and doxycycline (10 mg/kg SID). This study demonstrated a synergistic effect in the adulticidal activity of ivermectin and doxycycline when used in combination, implying that the use of doxycycline in heartworm treatment would be greatly beneficial in reducing the concentration of *Wolbachia* in all life-stages of *D. immitis* and improve adulticidal treatment efficacy.

With the high cost and occasional lack of availability of the traditional melarsomine canine heartworm treatment currently recommended by the American Heartworm Society, many clients and veterinarians are pushing for a more cost-effective option in treating canines. The American Heartworm Society’s recommended canine heartworm treatment utilizes 28 days of 10 mg/kg doxycycline BID, monthly macrocyclic lactone administration and the 3-dose protocol of melarsomine, administered on days 60, 90 and 91 respectively [5].

One slow kill protocol used by veterinarians for adulticidal canine heartworm treatment utilizes monthly topical administration of Advantage Multi®/Advocate® for dogs (10% imidacloprid + 2.5% moxidectin) (IMD + MOX) and administration of 10 mg/kg doxycycline BID for the first 30 days. While dogs treated with this slow kill protocol have been reported to have converted from heartworm antigen positive to negative, a definitive efficacy study for this treatment has not been performed. This study evaluates the efficacy of IMD + MOX and doxycycline in eliminating *Dirofilaria immitis* adults and microfilariae.

## Methods

A total of 16 beagles in two experimental groups, containing 8 dogs each, were utilized. The two experimental groups included the non-treated control dogs and treated dogs receiving Advantage Multi® for dogs (IMD + MOX) and doxycycline. All study dogs were purchased from a supplier with no previous exposure to any macrocyclic lactones. These dogs were all females and ranged from 17 to 34 months of age and 9.2–14.5 kg at the beginning of the study.

This laboratory study was conducted in accordance with VICH GL9 Good Clinical Practices (GCP), June 2000 (FDA Guidance for Industry 85, May 2001), the Study Protocol and UGA CVM Standard Operating Procedures.

Experimental heartworm infections were induced by surgical transplantation of *D. immitis* adult males and females [6]. Adult heartworms were harvested from donor dogs previously infected subcutaneously with 100 *D. immitis* infective third-stage larvae (L3) in the left and right inguinal region [7]. Transplanted adult worms were harvested from donor dogs at 10 months post-infection to ensure transplanted adults were sexually mature. A total of 11 adult females and 5 adult males were surgically transplanted into each study animal.

All study animals were randomized to experimental groups according to microfilarial counts approximately 30 days post-surgical transplantation of adult heartworms in descending order (highest to lowest). In the treated group, IMD + MOX was topically administered every 4 weeks for a total of 10 monthly treatments starting 4 weeks post-surgical transplant of adult heartworms. Dogs received either 1.0 ml or 2.5 ml IMD + MOX topical treatments according to the dog’s weight and the manufacturer’s recommendations. All dogs receiving IMD + MOX were weighed prior to each monthly topical treatment. Doxycycline was administered at 10.0–14.1 mg/kg (minimum dose of 10 mg/kg BID) for 30 days starting 4 weeks post-surgical transplant of adult heartworms.

During the administration of IMD + MOX and doxycycline or any other study activities in which possible exposure to products could occur, study personnel wore disposable gloves, aprons and boot covers. These items were changed and discarded between the two experimental groups. Whenever possible, daily activities for the non-treated control group were performed prior to activities for the treated group.

Blood samples were collected from each dog before surgical transplant of adult heartworms and every 4 weeks throughout the course of this study for *D. immitis* antigen testing, quantification of microfilariae, complete blood counts (CBC) and clinical chemistry profiles. Heartworm antigen tests utilizing the DiroCHEK® Heartworm Antigen Test Kit (Synbiotics Corporation, Zoetis, Kalamazoo, MI, USA) were performed according to the manufacturer’s recommendations and then read using a spectrophotometer (Epoch, BioTek Instruments Inc., Winooski, VT, USA) at a wavelength of 490 nm. Serum samples tested using the DiroCHEK® were quantified for the presence of heartworm antigen with and without heat-treatment. For the heat-treatment of samples, serum collected from whole blood was placed in a heat-block at 103 °C for 10 min and centrifuged at 14,000× *rpm* for 20 min [8]. The resulting supernatant was used for heartworm antigen testing in the DiroCHEK®. CBC and clinical chemistry profiles were analyzed by the University of Georgia’s College of Veterinary Medicine Clinical Pathology Laboratory.

In addition to heartworm antigen testing, blood samples were collected pre-surgery, on study days 1, 3, 7, 14, 21, 28 and then every 4 weeks thereafter to quantify circulating microfilariae throughout the course of this study. Thick smears were performed using heparin anti-coagulation blood collection tubes [7]. A modified Knott test was performed using heparin anti-coagulation blood collection tubes [7].

Euthanasia and necropsy were performed on study days 278–282. Dogs were sedated prior to being euthanized intravenously with Beuthanasia®-D solution (Intervet Inc., Merck Animal Health, Madison, NJ, USA). The heart, lungs and pulmonary vasculature and liver were removed and examined for gross pathology. Lesions of interest were examined histopathologically. Adult heartworms recovered were counted and sexed. Intact worms and fragments were counted and classified as decaying or viable. Total adult heartworm counts included the sum of the total intact worms per animal plus any identified non-degenerate fragments. Only non-degenerate adult worm fragments were included in the sum of total adult worms collected. If non-degenerate fragments could not be recognized as clearly from two separate worms then only 1 was added to the sum. Percent efficacy was determined by comparing the geometric mean worm counts of both the treated group and the non-treated control group using Abbott’s formula. Geometric means were calculated using a log + 1 method, to account for zero worm counts.

Statistical methods used to analyze microfilarial concentrations (thick smears and modified Knott) and the heartworm antigen (DiroCHEK) results involved a repeated measures analysis of covariance (RMANCOVA), with the best-fitted covariance structure, Satterthwaite’s adjustment for computing denominator degrees of freedom and using the Day 0 value as a baseline covariate. Adult heartworms recovered at necropsy were analyzed using an analysis of variance (ANOVA), after applying a logarithmic transformation of the heartworm counts. All analyses were performed using SAS® 9.3 software, utilizing an alpha level of 0.05 as significant.

## Results

**Fig. 1:**
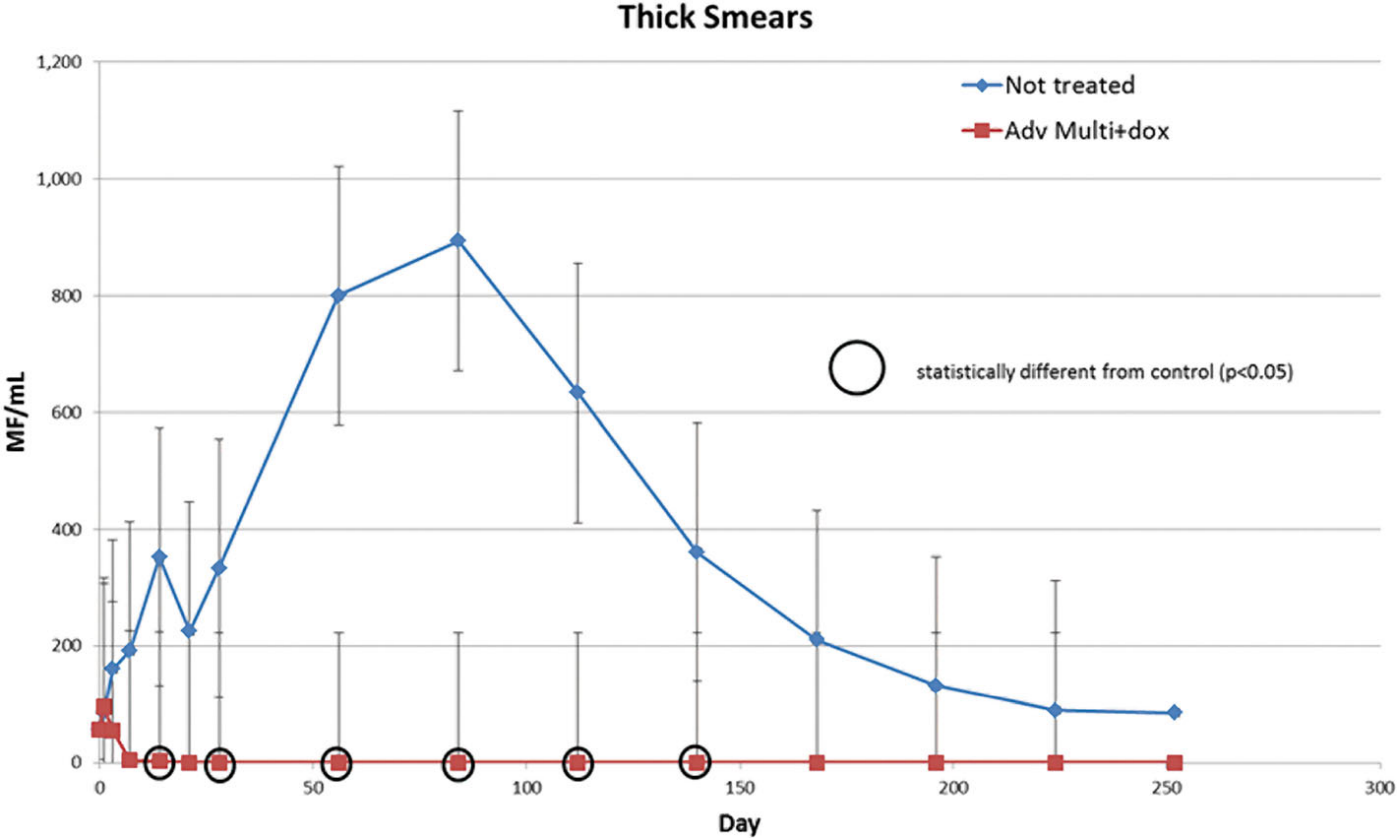
Microfilarial concentrations for the control and treated group calculated using thick smears throughout this study. All study dogs were evaluated for microfilarial concentration on study days 0, 1, 3, 7, 14, 21, 28 and every 4 weeks thereafter. The treated group exhibited significantly (*P* < 0.05) lower microfilarial concentrations compared to the control group on study days 14, 28, 56, 84, 112 and 140

With the initiation of IMD + MOX and doxycycline, microfilariae in the treated group declined and were no longer detectable by thick smears or modified Knott by 21 days post-treatment. No microfilariae were observed in the treated group throughout the remainder of the study. Microfilarial concentrations for all study dogs increased prior to the initiation of treatment in the IMD + MOX and doxycycline treated group. The numbers of microfilaria per ml were significantly lower (RMANCOVA, *t*-statistic ranging from 2.13 to 5.71, *df* = 59.4, *P*-values ranging from < 0.0001 to 0.0374) from dogs in the treated group as compared to the non-treated control dogs on study days 14, 28, 56, 84, 112 and 140 post-treatment (Fig. 1).

**Fig. 2:**
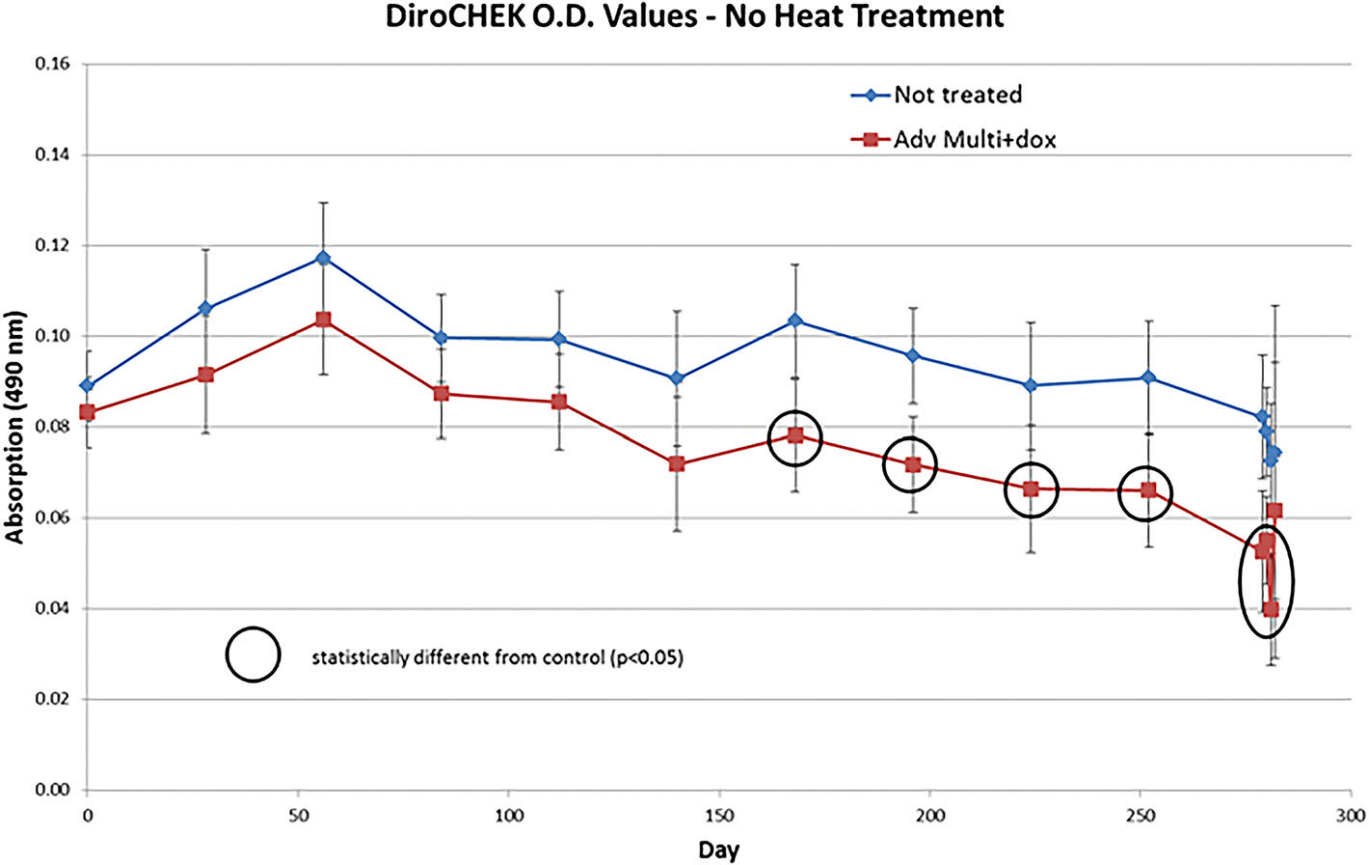
Optical density readings for the control and treated group throughout this study. Heartworm antigen concentration was obtained using the DiroCHEK® Heartworm Antigen Test Kit. Sample wells were read using on an ELISA at 490 nm to quantify absorption and the concentration of heartworm antigen present in each sample. Heartworm antigen concentration was measured every 4 weeks throughout this study. The treated group exhibited significantly (*P* < 0.05) lower optical density readings compared to the control group on study days 168, 196, 224, 252, 279, 280 and 281

The concentration of heartworm antigen was assessed using the DiroCHEK® Heartworm Antigen Test kit. All study dogs tested positive for the presence of heartworm antigen on study day 0 prior to the initiation of treatment. All non-treated control dogs remained positive for the presence of heartworm antigen throughout the entire study with very limited variability of optical density readings. Overall, the treated dogs began declining in the concentration of heartworm antigen present after 3 months of treatment. Additionally, there was an observed variability from month to month in the concentration of heartworm antigen detected. The optical density readings for the treated group was significantly lower than the control group from 6 months post-treatment throughout the remainder of the study (RMANCOVA, *t*-statistic ranging from 3.82 to 21.2, *df* ranging from 2.36 to 5.28, *P*-values ranging from 0.0036 to 0.0282) (Fig. 2). Serum samples collected at necropsy were tested for the presence of heartworm antigen pre- and post-heat-treatment. Both pre- and post-heat-treatment serum samples accurately detected either none or 1–2 adult worm infections as negative or positive respectively in the IMD + MOX and doxycycline-treated group (data not shown).

Throughout this study, dogs were not exercise-restricted during adult heartworm treatment. Pairs of dogs were housed in runs 4’W × 16’L. Only one adverse event occurred during this study. One study animal in the treated group presented with pale oral mucosa, rapid shallow breathing and lethargy, which was suspected to be the result of a pulmonary thromboembolism. No intervention was recorded and the event self-resolved within 24 h. This event occurred on study day 173, 5 days after the seventh monthly IMD + MOX topical treatment.

At necropsy, all personnel were blinded to the group and animal designation except for the recorder. The heart and lungs were removed from each animal and examined for the presence of adult heartworms. In the non-treated control group, adult heartworms were recovered from all 8 dogs ranging from 10 to 12 heartworms/dog with an average recovery of 10.6 adult heartworms/dog. In the treated dogs, no adult heartworms were found in 5 dogs, while 3 dogs had either one or two heartworms recovered, averaging 0.6 adult heartworms/dog. The overall treatment efficacy for the elimination of sexually mature adult heartworms using this treatment regimen is 95.9% (ANOVA, *F* = 130.28, *df* = 14, *P* < 0.0001) (Table 1).

**Table 1:** Efficacy of topical imidacloprid + moxidectin combined with oral doxycycline against transplanted adult *Dirofilaria immitis*. Adult heartworm recovery from necropsy for the control and treated groups. Necropsy was performed on study days 278–282, 10 months post-treatment

Group	Animal ID	No. of male worms recovered	No. of female worms recovered	Total^a^
Control	43493	3	7	10
	43491	4	6	10
	43486	5	6	11
	43481	5	6	11
	43492	3	7	10
	43480	5	7	12
	43489	4	6	10
	43484	4	7	11
Geometric mean				10.6^b^
Arithmetic mean ± SD				10.625 ± 0.74
Advantage Multi + Doxycycline Treated	43495	0	2	2
	43494	0	0	0
	43485	0	1	1
	43482	0	0	0
	43483	1	1	2
	43490	0	0	0
	43488	0	0	0
	43487	0	0	0
Geometric mean				0.44^b^
Arithmetic mean ± SD				0.625 ± 0.92
Treatment percent efficacy				95.9

^a^Total worm counts include intact worms plus non-degenerate fragments. Only 1 was added to the total worm number if non-degenerate fragments could not be identified as distinct worms

^b^The average recovery of adult worms for each group was calculated using the geometric mean of each group

Data from radiographs, echocardiograms, complete blood count, clinical chemistry profiles and necropsy pathology is to be published in a forthcoming manuscript.

## Discussion

The concentration of adult heartworm antigen began to decline 3 months post-treatment as detected by the DiroCHEK® heartworm antigen test kit. The marked peaks and troughs observed in the treated dogs were potentially indicative of adult worm death and subsequent increased release of heartworm antigen. While no necropsy was performed when each IMD + MOX and doxycycline treated dog tested negative for the presence of heartworm antigen, the DiroCHEK® accurately detected 1 and 2 adult worm infections as positive with and without heat-treatment at necropsy. This demonstrated that the heat-treatment of serum samples in this study of experimentally infected dogs was unnecessary.

This study demonstrates that the use of 10 monthly topical treatments of IMD + MOX every 4 weeks and 30 days of approximately 10 mg/kg doxycycline BID orally successfully eliminated circulating microfilariae and mature adult heartworms. The treatment efficacy of 95.9% using this treatment regimen is comparable to the melarsomine dihydrochloride 2-dose injection protocol. The administration of two 2.5 mg/kg injections 24 h apart resulted in a 90.7% efficacy in eliminating mature adult heartworms [9]. For dogs receiving the 3-dose injection protocol of one 2.5 mg/kg injection followed by the second and third injections 30 days after within 24 h apart, 100% of the adult male worms were eliminated while 98% of the adult females were eliminated. The overall efficacy of the 3-dose injection protocol is 99.0% [9].

As compared to other experimental alternative canine heartworm disease treatment protocols where the clearance of adult heartworms was low, the regimen defined in this paper eliminated more adult heartworms within a reasonable amount of time. The use of weekly prophylactic doses of ivermectin (6 μg/kg) orally for 34 weeks in combination with doxycycline (10 mg/kg/day) orally was able to eliminate circulating microfilariae by 12 weeks and had a 78.3% efficacy in eliminating mature adult heartworms after 36 weeks of treatment [4, 10]. Additionally, the use of ivermectin and pyrantel in 16 consecutive monthly treatments with Heartgard® Plus (6 μg/kg of IVM and 5 mg/kg of pyrantel, PYR, Merial) successfully eliminated microfilariae after 11 months of treatment and had a 56% reduction in mature adult heartworms [11].

In another experimental treatment regimen, 10 mg/kg doxycycline SID for 30 days and ivermectin and pyrantel (6–14 mg/kg) every 15 days for a total of 180 days was evaluated in naturally-infected heartworm-positive dogs. All treated dogs were negative for the presence of circulating microfilariae by day 90 and 72.7% of the treated dogs became heartworm antigen-negative by day 300. A necropsy was not performed to confirm treatment efficacy [12].

The high degree of efficacy observed with IMD + MOX and doxycycline at the short treatment duration of 10 months may be explained by the amount of moxidectin (minimum dose of 2.5 mg/kg) given during the study period compared to the lower labeled dosages of other macrocyclic lactones given in similar studies. One proposed mechanism for the increased efficacy of this treatment protocol is that the high level of exposure to moxidectin plays an important role in killing and elimination of the worms. A recent study evaluated the effect of IMD + MOX steady state and the efficacy in preventing canine heartworm disease [13, 14]. Previously, heartworm preventives were only thought to work backwards killing larvae entering the dog during the 30 days prior to product administration. However, the level of steady state moxidectin present in the treated dogs after four consecutive monthly doses was sufficient to prevent heartworm infection 28 days after the last IMD + MOX. The treatment regimen of IMD + MOX and doxycycline may be successful at eliminating sexually mature adult heartworms due to the concentration of moxidectin in IMD + MOX in addition to the steady state of moxidectin available in the body.

The risk of pulmonary thromboembolisms exists throughout any canine heartworm treatment. While only one treated dog presented with clinical signs indicative of a pulmonary thromboembolism, more of these events may have occurred during this study and were not clinically evident. Currently the American Heartworm Society recommends exercise restriction starting after the first melarsomine injection until 1 month after the last melarsomine injection. Therefore the recommended exercise restriction is for approximately 3 months. Since adult heartworm death using IMD + MOX and doxycycline is occurring over an extended period of time, the time period in which dogs have an increased risk of presenting with severe pulmonary thromboembolisms is longer. While dogs in this study were not exercise restricted, at this time we still recommend exercise restriction during the entire treatment regimen until the dog is antigen-negative.

## Conclusions

The treatment regimen of monthly administration of topical Advantage Multi® for Dogs (10% imidacloprid, 2.5% moxidectin) for 10 months and oral administration of approximately 10 mg/kg doxycycline BID for the initial 30 days of treatment was able to eliminate microfilariae within the first 21 days as well as mature adult heartworms over a shorter period of time as compared to other alternative treatment regimens utilizing macrocyclic lactones, therefore potentially minimizing the continued pathologic damage caused by adult heartworms during treatment. In conclusion, the treatment regimen in this study is a viable alternative when melarsomine dihydrochloride is not available, contraindicated or declined by an owner unable to afford more costly treatment or concerned about the potential side effects.

## Abbreviation

IMD + MOX: Advantage Multi® for Dogs (10% imidacloprid + 2.5% moxidectin) topical solution
